# Finding your feet: student participation during initiation of international clinical placements: Student participation during initiation

**DOI:** 10.1007/s40037-020-00561-9

**Published:** 2020-02-03

**Authors:** Miriam H. Wijbenga, Robbert J. Duvivier, Dale C. Sheehan, Stephan P. J. Ramaekers, Pim W. Teunissen, Erik W. Driessen

**Affiliations:** 1 European School of Physiotherapy/Center of Expertise Urban Vitality, Faculty of Health, Amsterdam University of Applied Sciences, Amsterdam, The Netherlands; 2 Parnassia Psychiatric Institute, The Hague, The Netherlands; 3 School of Health Professions Education (SHE), Faculty of Health, Medicine and Life sciences, Maastricht University, Maastricht, The Netherlands; 4 School of Medicine and Public Health, University of Newcastle, Newcastle, Australia; 5 College of Education, Health and Human Development, University of Canterbury, Christchurch, New Zealand; 6 Department of Obstetrics & Gynaecology, Amsterdam University Medical Centres, Amsterdam, The Netherlands

**Keywords:** Initiation, International placements, Student participation, Clinical workplace

## Abstract

**Introduction:**

International placements challenge students to find the right level of participation, as local practices, language and time pressure may affect their engagement in patient-related tasks or team activities. This study sought to unpack the initiation process during international clinical placements with the ultimate aim to achieve active student participation.

**Methods:**

Following a constructivist grounded theory approach, we conducted two individual interviews with 15 undergraduate healthcare students (before departure and whilst on placement). To identify emerging themes, we applied an iterative process of data collection and constant comparative analysis. Several team discussions informed further analysis, allowing us to reach a more conceptual level of theory.

**Results:**

From our findings we constructed a four-phase model of healthcare students’ initiation of international clinical placements, which brings into focus how the phases of ‘orientation’, ‘adjustment’ and ‘contribution to patient care’ build up towards a ‘sense of belonging’. We identified several factors that induced active student participation in practice, such as a favourable workplace setting, opportunities for learning and a local support network.

**Discussion:**

Active student participation is aimed at different goals, depending on the four phases of initiation that eventually lead to a sense of belonging and support workplace learning.

**Electronic supplementary material:**

The online version of this article (10.1007/s40037-020-00561-9) contains supplementary material, which is available to authorized users.

## Introduction

Nowadays, it is common for healthcare students from different disciplines to engage in international placements, a learning experience that exposes them to a wide variety of workplace settings. To maximise this experience, students must learn to cope with and adjust quickly to changing circumstances. Consequently, it is widely assumed that international placements can foster students’ development into professionals that are ‘fit for practice’: who are open to ongoing developments, able to deal with change in their respective disciplines and ready to face future healthcare challenges. According to the concept of ‘legitimate peripheral participation’ [1, 2], students undergo a process of realignment with their social learning environment when entering practice [3]. Research on workplace learning suggests that this realignment, in addition to being highly influenced by context [4], is a result of students’ engagement in novel activities [5] and support provided by the supervisor or local healthcare team [6, 7]. It is therefore important that students find ways to engage in the clinical workplace, especially during international placements where different criteria and expectations regarding professional practice might apply [5, 6]. Currently, there is a lack of knowledge of what helps and hinders students in engaging in and learning from their work during international placements.

Students face opportunities and challenges when starting a new clinical placement [3, 8]: they step out of their familiar educational framework and into a different frame of professional practice. This transition into the workplace can be stressful, even if it is not in a foreign country, as students may struggle to understand their role and responsibilities in the clinical setting. Besides being newcomers to the workplace, students are unfamiliar with local rules, professional hierarchies and social interactions [2, 9]. Such uncertainties may be more profound when entering an international setting, where language barriers or cultural differences may negatively affect professional interaction between the student and local healthcare team [10]. Affordances of practice such as opportunities for learning or ‘developmental space’ [2] may be less obvious in international clinical settings, thereby inhibiting student participation.

Previous research by Sheehan, Wilkinson and Billet [11] indicates that during the initiation phase of workplace learning it is imperative that the learner feels invited to participate and engages with the team. When arriving in a new learning environment or workplace several factors related to team organisation, supervision and student behaviours can support this initiation to practice, yet it is unclear how these social processes develop. The authors introduced a model on intern participation in medical workplaces explaining how different elements should lead towards a more ‘stable’ maintenance phase where actual workplace learning can occur [11]. It is unknown if this model holds true for the allied health professions. Furthermore, students entering placements in an international context have different needs and expectations than locally trained students [10, 12, 13], including a clear need for preparation [14]. This mindset, starting at preparation, further contributes to the challenges faced when initiating international clinical placements.

Our study focuses on the interplay between these individual needs and expectations regarding practice during international clinical placements and the workplace learning conditions existing in such settings. By investigating student participation in unfamiliar cultural and geographical contexts, we aim to gain insight into the different factors involved in the initiation phase of international clinical placements.

## Methods

This qualitative study followed a constructivist grounded theory approach [15], using existing frameworks as sensitising concepts to investigate the process of initiation of international clinical placements. We applied an iterative process of data collection and constant comparative analysis, alternating with several rounds of team discussion. This approach allowed us to learn how student participants experienced initiation of placement as well as explore the underlying mechanisms and conceptualise our findings into theory ‘grounded’ in data.

### Setting and participants

Undergraduate healthcare students at Amsterdam University of Applied Sciences (AUAS) undertake regular 10- to 20-week clinical placements as part of their professional training. Only a few go on an international placement, apart from the students at the European School of Physiotherapy (ESP), who undertake at least three out of four clinical placements abroad. The AUAS internship office offers support in procuring international placements. During placement, students receive both home-based support from a staff member and local, one-to-one supervision. We purposively sampled all Year 2 Physiotherapy and Exercise Therapy students planning to undertake a 10- to 20-week international placement starting in November 2017 or February 2018. Among these, the ESP students had already completed an internship abroad. We sent an informative, personal email to 25 students who had successfully completed their first placements, inviting them to participate in our project. Twelve female and three male students (Physiotherapy [*n* = 5], Exercise Therapy [*n* = 3] and ESP [*n* = 7]) volunteered to participate (Tab. 1) after signing informed consent. Placements took place in Europe (*n* = 4), Asia (*n* = 2), Australia (*n* = 1) Africa (*n* = 3), North America (*n* = 1) and South America, including the Caribbean (*n* = 4).

**Table 1:** Participant details

Student	Gender	Setting	Placement region	Organised by
ESP1	F	Hospital	Europe	University
ESP2	M	Private practice	Europe	University
ESP3	M	Private practice	Asia	Student
ESP4	F	Hospital	Africa	University
ESP5	F	Private practice	North America	Student
ESP6	F	Hospital	Africa	University
ESP7	F	Private practice	Africa	University
PT1	F	Hospital	Europe	University
PT2	F	Hospital	Europe	University
PT3	F	Rehabilitation centre	Asia	University
PT4	M	Private practice	Australia	University
PT5	F	Private practice	South America	University
ET1	F	Private practice	South America	University
ET2	F	Private practice	South America	University
ET3	F	Private practice	South America	University

### Data collection

We conducted two individual interviews between October 2017 to March 2018: one before departure and one when students had undergone a month of placement, allowing ample time for initiation. Semi-structured interview guides were developed and tested (see Appendix A of the Supplementary Electronic Information). The questions focusing on students’ preparation and their experiences were initially informed by the model of Sheehan, Wilkinson and Billet [11]. Questions were further developed based on constant comparative analysis, informing subsequent data collection. Due to varying start and end dates of placements within the different programmes ESP students were interviewed first. MW conducted all interviews in person or via Skype, averaging 30 minutes for the first interview (range 24–44 minutes) and 45 minutes for the second (range 34–55 minutes).

### Data analysis

For this exploratory study, we adopted a constructivist grounded theory approach [16, 17], following an iterative process of simultaneous data collection and analysis. All interviews were audio recorded, transcribed verbatim and anonymised. Two researchers (MW and RD) open-coded the first set of transcripts independently, before discussing initial findings with the research team (MW, RD, DS and PT). The outcome of these discussions informed subsequent data collection during round 1. Further analysis led to adaptations to the second interview guide, particularly more focus on restrictions of student participation, level of independence and collaboration, as this seemed to be central to students’ preparation. Transcript summaries were shared with all participants, who agreed that they reflected their views without further additions. MW and RD then applied axial coding to all remaining transcripts in order to define connections between categories, refining and regrouping them into overarching themes. After having established consensus on the categories identified, they again shared their findings with the full research team, using a process of constant comparative analysis through memo writing to further develop the emerging theory. Consequently, the team discussion was able to reach a more abstract level of theory that remained ‘grounded’ in the data. We used ATLAS.ti v8.3.1 to support the analysis.

## Results

**Fig. 1:**
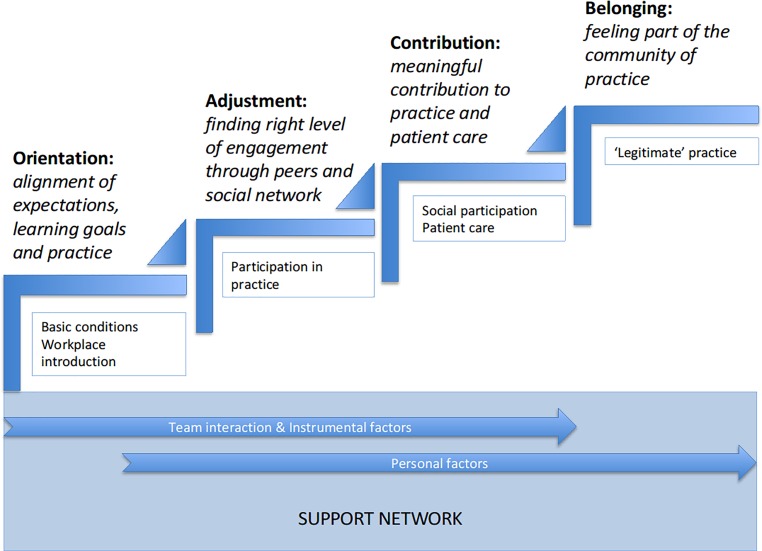
Four-phase model of healthcare students’ initiation of international clinical placements

Interview data combined into four overarching themes, resulting in the model of healthcare students’ initiation of international clinical placements, as shown in Fig. 1. This model presents the different phases of initiation that build towards a sense of ‘belonging’ over time. Partially overlapping, these phases are influenced by several factors that we will explain in the following. To elucidate our findings, we have included illustrative quotes throughout the Results section, with participant information in brackets (nationality, gender, age and continent of placement).

### Orientation

Students described how initiation of clinical practice in an international context was intertwined with tackling the general challenges of going abroad. Several students highlighted the importance of arranging basic conditions such as accommodation, transport and food prior to placement so they could focus on their placement experiences. Often students consulted peers who had visited the area or clinic before, which helped their orientation and provided access to local contacts. Balancing workplace conditions such as a demanding work schedule with personal challenges in a new living environment felt highly challenging for some:

*You are continuously busy and you don’t really have time to say ‘I can review in the evening’. Working hours are between ten and six o’clock. After six o’clock you might be able to study for a little while, but we live half an hour away by bus; a three quarters of an hour walk. … It was quite a problem. (Dutch female, 21 years old, Asia)*

In the first interview round, students explained that orientation focused on clarifying mutual expectations regarding affordances of practice during the international placement. To achieve this, some students proactively contacted the team they were going to join via email or Skype to get information about workplace-related preparation and learning potential:

*We had been emailing. But it was really nice to talk to him [via Skype], because he made his expectations very clear and I told him, well, ours, which he knows already. He basically said that for him it’s a learning experience for me, which is a really nice way to give me space. He said that what I put in, I’ll get out, type of thing. (Swiss female, 25 years old, Africa)*

Discussing previous placement experiences helped identify potential strategies for learning and the level of support needed when entering the workplace. Students who had difficulties communicating before departure felt less prepared to enter practice in their new learning environment:

*… the clinical instructor unfortunately is very slow in responding at this moment, although that may have to do with the clinical circumstances, I presume …, which means I am going abroad on a clinical placement but have no idea what to expect. I am also left with a lot of questions. (Dutch female, 20 years old, Europe)*

Students would often be welcomed to their placement by an experienced therapist responsible for supervision. In most cases, this clinical instructor had also been their first contact person, who was now introducing them further to the workplace and the team, explaining local rules of practice and clarifying mutual expectations:

*Yes, I do believe it was kind of a standard talk. [As new interns] we were able to ask questions as well. But it was all very relaxed, like: ‘This is what you’ll be doing, and you will … You can choose someone to supervise you and work with. And slowly but surely you will grow into your own patient load’. (Dutch female, 20 years old, Europe)*

Regardless of their preparation, however, students could still be personally challenged by the new and unfamiliar context, for instance when encountering cultural differences with respect to their professional position:

*We must keep a certain distance from the clients. Because here, even if you look them in the eye or look at them, they may think you are flirting with them. And that’s not what you want in here. So, I feel I have a professional role and want to keep it that way, which means I have to be careful somehow. (Dutch female, 20 years old, Asia)*

Once expectations, learning goals and local practices were aligned, students became more comfortable to start participating in patient-related activities:

*Also when I was talking to the local physiotherapy students, [I realised] it is pretty much the same how you structure your sessions, for example in patient history and what registration forms you use, and, yes, how you are supposed to do your assessment. (German female, 22 years old, Africa)*

### Adjustment

During the second phase all elements seemed to be in place for the student to begin workplace learning. Students started engaging actively once individual learning goals had been aligned with the clinical environment:

*At the beginning I found it difficult to be engaged … I found it difficult to remember what they were testing and why and then the different steps. So, I became more engaged as time passed I would say. (Swiss female, 25 years old, Africa)*

Generally, having peers around in the clinical workplace facilitated students’ adjustment to the clinic. Students reported that they felt more at ease, as peers provided a safety net and someone to share their experiences. Consequently, students felt more confident to engage. At the same time, the presence of peers could also inhibit student engagement:

*I think it’s just inevitable … If you see a new person around, it’s easier to kind of engage them if they’re on their own. And it’s just like, when there’s a group it’s much more effort or whatever, they’re all … Like, say that the surgical team wanted to invite you along to something, then it’s like, well, there’s three of them, so probably not. Whereas if it was just one, it probably would happen. (British female, 27 years old, Africa)*

Students explained how other healthcare professionals also became part of their local support network. Shared communal treatment areas, such as a gym, where professionals worked alongside each other, invited more active student participation. Students saw less opportunities for social interaction with the team, however, when they worked closely together with their clinical instructor in smaller private practices or received patients in separate rooms, leaving little exposure and contact time with other professionals:

*In [the main location] there are only separate rooms whereas in [the other location] there’s the gym where almost every therapist is working. It’s just a large space with different beds, closed off by curtains, which makes it more accessible. Because you can simply ask: ‘look, I don’t know exactly, could anyone please help me out with this test?’ or something. (Dutch male, 24 years old, Australia)*

Throughout the adjustment phase proactive behaviours, such as asking questions and engaging with the team, appeared key to successful participation. Positioning yourself within the clinic and judging the right level of participation seemed a rather complicated task in practice, as it required not only formal knowledge of team organisation and policies, but also tacit knowledge:

*[As a new intern] I do not want to take too much space. But I also want to learn of course, so some kind of space I have to take. So yes, it is this thing of trying to understand with each person, how is it that you make it harmonious, the situation, without breaking the flow of the session, also because there is a patient there. (French male, 33 years old, Europe)*

### Contribution to patient care

Before being able to make a meaningful contribution to practice and patient care, students reflected on how they could first arrive at a sense of belonging to the team and the service in a social, ‘community of practice’ sense. This involved trying to find out about the ‘informal’ rules of practice and how to balance the roles of participant versus observer. Seeing and taking opportunities for learning required self-confidence and flexibility as well as proactive behaviour. Several students stated it was not always clear to what extent clinical instructors, team members or even patients allowed them to ‘poke their nose in’. Moreover, they added that this ongoing ‘negotiation’ of practice demanded considerable energy, which was not always available:

*I think it’s important to gauge the energy levels of the physiotherapist to see how much they want to involve me or not. So, I succeed sometimes, other times I am not engaged because I am tired. And then other times I am not engaged because I … yes, I feel like it would kind of rock the boat too much. (Swiss female, 25 years old, Africa)*

Often, the clinical instructor played an important part in helping the student become a ‘legitimate’ participant, by engaging them in patient-related activities, expressing confidence or giving responsibility, thereby inviting them to participate:

*Well she said that we are very independent, and that we get our patients and then … most of the time we take the patients to the physiotherapy room and try to treat them. Once you are kind of done with your patients, you are usually free or you can do something else, so yes, that is what they’ve told us. (German female, 22 years old, Africa)*

Finally, students felt that language barriers sometimes negatively affected team engagement or patient communication, directly impacting their level of participation, especially as communicating in a foreign language could feel taxing and inhibit social interaction with the team:

*So then there will be not everyone, but five, six physiotherapists that have lunch together. In these meetings for example, everything happens in a foreign language and I understand not everything but quite some. But then it is again … Like I do not want to- If I start speaking in English then the whole situation changes to English and I feel like I [snaps fingers] break the flow or something? But that is more me. (French male, 33 years old, Europe)*

### Belonging

Within the first month of placement, all interviewees learned how to approach professional challenges faced in the international clinical setting and to turn personal insecurities into professional development opportunities. They were able to reflect on how the initiation process helped clarify their position as a learner within the clinical workplace. In the end, the international placement experience contributed to their self-confidence as a professional as well as to their level of independence:

*My biggest personal goal was to become more self-confident and speak up in case people would want me to simply follow their lead, which I usually allow. And now I felt I really had stood up for myself, so I can proudly say that I have managed the situation well. And I think, if I hadn’t done so, I might have ended up feeling a lot less comfortable [in this placement] than I do now. (Dutch female, 21 years old, Europe)*

Ultimately, students felt able to make a meaningful contribution to patient care: having become part of the community of practice themselves, they had now reached a more stable state of participation, or: ‘found their feet’ (Swiss female, 25 years old, Africa). By this time, the students experienced a sense of belonging in that they had gained a voice, contributing knowledge to local practice and providing input to—not just learning from—the team:

*I remember before I had my first own patients, I would be in co-therapy and I would barely say anything and barely speak to the patient and just follow instructions, basically. Now, when I’m in with a therapist, I also communicate with the patient. Also, when they give me the space to lead, I’m no longer [too] shy to take over and to act. (German female, 22 years old, Europe)*

## Discussion

Initiation of international clinical placements follows four different phases: orientation, adjustment, contribution and belonging. During the *orientation* phase (phase 1), students must make practical preparations to meet their basic needs in and outside the workplace, allowing them to direct their energy towards their learning process. During the *adjustment *phase (phase 2), students require space and time [2] in order to adjust to the workplace setting and local team, before feeling comfortable enough to start participating in practice [7]. Part of this links with getting to know the ‘informal’ rules of practice [18] to a point where students can read and anticipate the social cues in the new culture. To achieve this, students not only must learn about their new physical environment, tasks and interaction and communication with patients and the team [19], but also acquire cultural sensitivity, which is challenging in a different culture. By *contributing *to patient care and team activities (phase 3), students come to identify opportunities for learning [4, 20] and build confidence, indispensable for creating a sense of belonging to their new ‘community of practice’ [5, 11]. In order to become full or ‘legitimate’ participants [1] and reach a true sense of *belonging* (phase 4), students seemingly must take another step forward and position themselves as healthcare professionals rather than interns [21].

Building on the ideas of Lave and Wenger [1] and Sheehan, Wilkinson and Billet [11], our model depicts four stages that provide a more detailed understanding of clinical practice initiation. Although previous research has addressed similar concepts about preparation, in-country experiences and reflection/debriefing upon return [14], our model focuses on the transition into the clinical workplace, and therefore the start of workplace learning. By investigating the context of allied healthcare we have broadened the scope of previous theories [7, 9], and further refined an established model of workplace initiation [11].

Both personal and external factors play an important role in student transition between the different phases towards the end goal of ‘belonging’ where true workplace learning can occur [22]. This does not necessarily imply that all students always go through these phases in this particular order or are capable of reaching this final step, especially during relatively short engagements. Potential changes in working conditions, such as ward rotations during placements, may set students back from the adjustment phase as they have to discover new ‘informal’ rules of practice [18], whilst the supervisory team may already be expecting a different level of participation. Students may also become ‘stuck’, for example during orientation when practical hitches such as time restrictions or poor Internet connectivity thwart communication.

Although we did not investigate how much time students need to successfully complete these different phases of initiation, short-term placements often offer too little time to discover the hidden rules of engagement [23] and may result in students remaining ‘tourists’ rather than becoming ‘sojourners’, as described by Wenger [24]. However, our model delivers a conceptual framework to better understand the challenges faced during initiation, whilst pointing out potential areas for ‘repair’ in the different phases. The results of our study, moreover, confirm that students are likely to engage in practice once they have a clear position and supported role [6, 7] and that they are less likely to contribute to patient care [12, 25, 26] and have a sense of belonging when facing difficulties in communication, time constraints, lack of practical guidelines and unfavourable supervisor and learner behaviours. This aligns with an article by Dornan and colleagues [27] who emphasise the importance of challenging students to step outside their comfort zones, supporting students’ learning by organising the learning environment in a way that invites them to participate in practice.

When the aim is to enable workplace learning through active student participation, short international placements seem to be a risk [7]. A review by Atherley and colleagues underscribes the need to “combine elements of educational, social and developmental perspectives” to smoothen transition [28]. Accordingly, home institutions could help students prepare for local circumstances by engaging them in an early stage, preferably before their placement, thereby supporting students’ orientation phase. Our study confirms that relationships within the local support network, including the clinical instructor, healthcare professionals, peers or even family members, could play an important role in accommodating students in and outside the clinic during placement to support the initiation process [28]. Additionally, our study revealed how important it is for students in an international context to be proactive and show their willingness to engage, especially to the clinical instructor, although this may be affected by cultural sensitivity [13]. These findings raise questions about the purpose and learning goals of short international placements or medical electives, suggesting a need for future research into the impact of the initiation phase on overall placement experiences.

### Strengths and limitations

One strength of our study was that it involved multiple disciplines within the Faculty of Health at AUAS in the Netherlands. Moreover, it included international healthcare student placements in locations worldwide spanning a wide range of clinical settings. Due to varying start and end dates of the placements within existing curricula we conducted interviews in two waves, which limited the traditional iterative approach of constructivist grounded theory. Since all participants were volunteers, selection bias may have occurred towards those who consciously prepared for their placement. We used semi-structured interviews to inquire after self-perceived initiation instead of actual workplace initiation.

## Conclusion

Active student participation starts upon initiation of international placements, or even before the students’ arrival. This participation, however, is aimed at different goals, depending on the four phases of clinical practice initiation that eventually lead to a sense of belonging and enhance workplace learning. The results of this study question the value of short-term placements such as international electives, given the time students need to fully utilise the workplace learning opportunities afforded by the placement. Understanding the different phases of initiation can help support students in adjusting and moving to full participation and belonging.

## Caption Electronic Supplementary Material

Appendix—Interview guides
