# Supplementary material for: Finding your feet: student participation during initiation of international clinical placements: Student participation during initiation
Source: Perspect Med Educ. 2020 Feb 3;9(1):41–8. doi: 10.1007/s40037-020-00561-9 (PMC7012961; doi:10.1007/s40037-020-00561-9)
Supplement: Supplementary file 1 — Appendix—Interview guides [file 40037_2020_561_MOESM1_ESM.docx]

***Appendix - Interview guides***

**Interview 1 (preparation phase)**

*Opening questions*:

Where will you go to during your next internship? Are you looking forward to it? Was there a particular reason for organising this international internship during this period?

*Preparation of clinical internship*:

1. How did you prepare yourself for this internship? Whom did you consult for information? Did you take any courses or precautions?
2. What do you know about the healthcare setting where your internship will take place? Where did you get this information? Is there anything else you would like to know about the clinical setting (e.g. team roles, work schedule, expectations, etc.)?

*Student expectations*:

1. How do you think the local healthcare team will receive you? How/by whom will you be introduced to the workplace?
2. What do you know of the local rules and regulations regarding physiotherapy/occupational therapy/the healthcare system in <country>? How/where did you find this information?
3. What kind of work activities do you expect to become involved in during this internship? What level of independence do you expect?
4. What do you think your clinical instructor/healthcare team/peers expect of you in order to make your internship a successful international experience?

**Interview 2 (follow-up after first month):**

*Opening questions*:

How have you experienced your internship so far?

*Preparation of clinical internship*:

1. How have your preparations for this internship supported your adjustment to this clinical setting? Have you missed any information when preparing for this internship?
2. Has the healthcare setting met your expectations? In what way?

*Student expectations*:

1. How have you been received by the local healthcare team? Who has introduced you to practice? What did this introduction entail?
2. How do local rules and regulations influence your ability to actively participate in practice? How does this influence your engagement with the team?
3. Has the internship met your expectations so far? What work activities have you been involved in and to what level of independence? In what way does your work differ from clinical practice in the Netherlands?
4. Can you describe a practical situation that was significant to understanding your position as an intern within this international clinical setting?
